# T1 mapping by CMR in patients with arrhythmic cardiomyopathy

**DOI:** 10.1186/1532-429X-17-S1-P266

**Published:** 2015-02-03

**Authors:** Lei Zhao, Jing An, Tianjing Zhang, Xiaohai Ma, Zhanming Fan

**Affiliations:** Beijing Anzhen Hospital, Beijing, China; MR Collaborations NE Asia, Siemens Healthcare, Beijing, China, Beijing, China

## Background

Traditionally, late gadolinium enhancement (LGE) helps effectively detect focal fibrosis in cardiomyopathy. If patients have diffuse fibrosis, LGE could not provide enough objective evaluation of the severity of the disease. Arrhythmic cardiomyopathy (ACM) is a clinical entity which corresponds to all myocardial changes induced by chronic tachycardia. For ACM patients, myocardial fibrosis evaluation is important for treatment and prognosis. Accordingly, we aimed at exploring associations between myocardial T1 changes as a surrogate for left ventricular (LV) interstitial fibrosis in ACM.

## Methods

Twenty-one patients with ACM underwent cardiac magnetic resonance (CMR) before catheter ablation. 19 healthy volunteers served as control group. CMR imaging was performed at 3.0T (Siemens MAGNETOM Verio) including cine, pre- and post-contrast T1 mapping, and LGE imaging. T1-maps were obtained in the same short-axis planes of LGE imaging before and 15minutes after the administration of gadolinium by using a modified Look-Locker (MOLLI) sequence. T1 times in 3 short axis slices (basal, mid-ventricular and apical) were evaluated.

## Results

ACM patients had the same age as control group (46.2±17.6 vs. 43.8±14.5, p=NS). Mean pre-contrast T1 time, post-contrast T1 time and reduced T1 time were significantly different in patients with ACM compared with control groups (1364.0±66.6 vs. 1292.9±69.0ms, 485.3±50.7 vs. 581.4±61.7ms, 878.7±97.0 vs. 624.9±166.4ms, all p<0.01). When the analysis was restricted to ACM patients without late gadolinium enhancement (LGE, n=16), these differences remained significant (figure).

**Figure 1:**
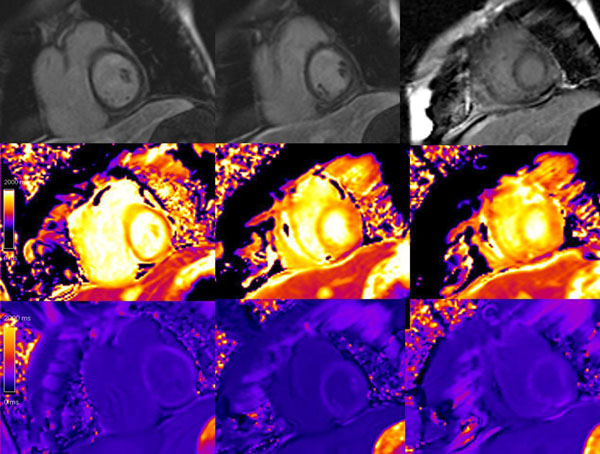
Arrhythmic cardiomyopathy patient's LGE and T1 mapping images. Upper lane are the LGE images in the basal, mid-cavity, and apical level, middle lane are the pre-contrast T1 mapping images, lower lane are the post-contrast T1 mapping images. No focal delayed enhancement lesions were found in the LGE images. The mean T1 value of pre-contrast images is 1430.6 ms, the mean T1 value of post-contrast images is 434.6 ms.

## Conclusions

Arrhythmic cardiomyopathy patients' myocardium show increased pre-contrast T1 times, decreased post-contrast T1 times and increased reductions in myocardial T1 times compared with healthy person. Abnormal myocardial T1 times, a surrogate of diffuse interstitial fibrosis, are found in arrhythmic cardiomyopathy.

## Funding

Supported by the China National Natural Science Fund Grant (81101173).

